# Multi-trait genome-wide association study of opioid addiction: *OPRM1* and beyond

**DOI:** 10.1038/s41598-022-21003-y

**Published:** 2022-10-07

**Authors:** Nathan Gaddis, Ravi Mathur, Jesse Marks, Linran Zhou, Bryan Quach, Alex Waldrop, Orna Levran, Arpana Agrawal, Matthew Randesi, Miriam Adelson, Paul W. Jeffries, Nicholas G. Martin, Louisa Degenhardt, Grant W. Montgomery, Leah Wetherill, Dongbing Lai, Kathleen Bucholz, Tatiana Foroud, Bernice Porjesz, Valgerdur Runarsdottir, Thorarinn Tyrfingsson, Gudmundur Einarsson, Daniel F. Gudbjartsson, Bradley Todd Webb, Richard C. Crist, Henry R. Kranzler, Richard Sherva, Hang Zhou, Gary Hulse, Dieter Wildenauer, Erin Kelty, John Attia, Elizabeth G. Holliday, Mark McEvoy, Rodney J. Scott, Sibylle G. Schwab, Brion S. Maher, Richard Gruza, Mary Jeanne Kreek, Elliot C. Nelson, Thorgeir Thorgeirsson, Kari Stefansson, Wade H. Berrettini, Joel Gelernter, Howard J. Edenberg, Laura Bierut, Dana B. Hancock, Eric Otto Johnson

**Affiliations:** 1 GenOmics, Bioinformatics, and Translational Research Center, Biostatistics and Epidemiology Division, RTI International, Research Triangle Park, NC USA; 2 The Laboratory of the Biology of Addictive Diseases, The Rockefeller University, New York, NY USA; 3 Department of Psychiatry, Washington University School of Medicine, St. Louis, MO USA; 4 Dr. Miriam and Sheldon G. Adelson Clinic for Drug Abuse, Treatment and Research, Las Vegas, NV USA; 5 Genetic Epidemiology, QIMR Berghofer Medical Research Institute, Brisbane, Australia; 6 National Drug and Alcohol Research Centre, University of New South Wales, Randwick, NSW Australia; 7 Institute for Molecular Bioscience, The University of Queensland, Brisbane, QLD Australia; 8 Medical and Molecular Genetics, Indiana University School of Medicine, Indianapolis, IN USA; 9 Department of Psychiatry, State University of New York Downstate Medical Center, Brooklyn, NY USA; 10 SAA-National Center of Addiction Medicine, Vogur Hospital, Reykjavik, Iceland; 11 deCODE Genetics/Amgen, Reykjavik, Iceland; 12 Department of Psychiatry, University of Pennsylvania, Philadelphia, PA USA; 13 Department of Psychiatry, Yale University School of Medicine, West Haven, CT USA; 14 School of Psychiatry and Clinical Neurosciences, The University of Western Australia, Perth, WA Australia; 15 School of Population and Global Health, Population and Public Health, The University of Western Australia, Perth, WA Australia; 16 Hunter Medical Research Institute, Newcastle, Australia; 17 School of Medicine and Public Health, The University of Newcastle, Callaghan, NSW Australia; 18 School of Biomedical Sciences and Pharmacy College of Health, Medicine and Wellbeing, The University of Newcastle, New Lambton Heights, NSW Australia; 19 Faculty of Science, Medicine and Health, University of Wollongong, Wollongong, NSW Australia; 20 Department of Mental Health, Bloomberg School of Public Health, Johns Hopkins University, Baltimore, MD USA; 21 Department of Family and Community Medicine, Saint Louis University, Saint Louis, MO USA; 22 Faculty of Medicine, University of Iceland, Reyjavik, Iceland; 23 Department of Psychiatry, Genetics, & Neuroscience, Yale University School of Medicine, West Haven, CT USA; 24 Department of Biochemistry and Molecular Biology, Indiana University School of Medicine, Indianapolis, IN USA; 25 Department of Psychiatry, Washington University, St. Louis, MO USA; 26 Fellow Program, RTI International, Research Triangle Park, NC USA; 27 Genome Science Institute, Boston University, Boston, MA USA

**Keywords:** Genetic association study, Haplotypes

## Abstract

Opioid addiction (OA) is moderately heritable, yet only rs1799971, the A118G variant in *OPRM1*, has been identified as a genome-wide significant association with OA and independently replicated. We applied genomic structural equation modeling to conduct a GWAS of the new Genetics of Opioid Addiction Consortium (GENOA) data together with published studies (Psychiatric Genomics Consortium, Million Veteran Program, and Partners Health), comprising 23,367 cases and effective sample size of 88,114 individuals of European ancestry. Genetic correlations among the various OA phenotypes were uniformly high (r_g_ > 0.9). We observed the strongest evidence to date for *OPRM1*: lead SNP rs9478500 (*p* = 2.56 × 10^–9^). Gene-based analyses identified novel genome-wide significant associations with *PPP6C* and *FURIN*. Variants within these loci appear to be pleiotropic for addiction and related traits.

## Introduction

In 2020 the US recorded the highest 12-month count of opioid overdose deaths, > 70,000^1^, which represents a 40% increase since 2019, a > 250% increase since 2000^2^, and is 1.7 times the number of deaths caused by automobile crashes in 2020^3^. Approximately 4% of the US population aged 12 and older (10.1 million people) misused opioids in 2019, with 1.6 million people initiating new prescription opioid misuse^4^. The most recent annual estimate of the total economic burden of prescription opioid abuse and dependence in the US (2013) is over $78 billion^5^, including Medicaid spending of more than $8 billion on opioid addiction (OA) treatment^6^. By every metric, the opioid epidemic continues to be a tremendous burden, and the need to expand the medication-assisted treatment toolkit for OA through identification of new targets for drug development is clear^7^.

Animal model and human neuroimaging studies have established a strong, albeit partial, understanding of the neurocircuitry of addiction as heuristically characterized in the Koob and Volkow model^8^. The primary neurocircuitry elements involved (basal ganglia, extended amygdala, and prefrontal cortex) and their molecular connections to the cycle of addiction (intoxication, withdrawal, and preoccupation) are broadly understood. However, there is clear variability in the functioning of this neurocircuitry among individuals as evidenced by only 20–30% of people who use heroin becoming addicted^9,10^ and only 8–12% of chronic pain patients prescribed opioids developing OA^11^.

Genetics is a major contributor to individual variation in the risk of developing OA, with ~ 60% of the population variability being attributable to genetic factors^12,13^. This heritability estimate is comparable to other complex phenotypes, such as Alzheimer’s^14^, age-related macular degeneration^15^, and height^16^, which have conclusively associated genetic variants. However, few robust genetic variant associations with OA have been identified^17–20^.

Eight genome-wide association studies (GWAS) of OA have been reported^21–28^, in which the number of cases varied from 104 to 10,544 for ancestry specific analyses. Six of these GWAS identified genome-wide significant (GWS) loci^23,25–29^. However, only the largest analysis, which combined results of European ancestry (EA) cohorts from the US Veterans Affairs Million Veteran Program (MVP), the Study of Addiction: Genetics and Environment (SAGE), and Yale-Penn (YP) cohorts (10,544 cases and 72,163 controls), identified a GWS association that replicated in an independent sample (additional YP data: 508 cases and 206 controls). The variant identified is the long-studied rs1799971 (*OPRM1*-A118G), a functional coding variant (encoding Asn40Asp) in the mu opioid receptor gene (*OPRM1*): discovery *p* = 1.51 × 10^−8^, replication *p* = 0.049. The rs1799971-G protective association with OA was also extended at nominal significance to buprenorphine treatment status in the UK Biobank (240 cases and 360,901 controls; *p* = 0.04).

To maximize discovery, we leveraged genomic structural equation modeling (gSEM)^30^ to combine new and existing GWAS data with varied, but closely related, phenotypes for OA to enable the largest GWAS of OA to date (23,367 cases, 384,629 controls: effective sample size 88,114). We brought together novel results from the Genetics of Opioid Addiction Consortium (GENOA) with publicly available summary statistics from the MVP-SAGE-YP^27^, the Psychiatric Genetics Consortium-Substance Use Disorder Group (PGC-SUD)^26^, and the Partners Health Group (PH)^28^. We examined SNP-based heritability and genetic correlation among the varied phenotypic definitions of OA across the contributing cohorts, including diagnostic and frequency of use-based cases and different types of controls: opioid exposed, unexposed, and population-based. We conducted a variant level gSEM analysis in the full complement of cohorts and a gene-based association test based on those results. Whereas gSEM accounts for the sample overlap among the GENOA, PGC-SUD, and MVP-SAGE-YP analyses, we were able to combine the samples and increase substantially available sample size compared to standard meta-analysis. Follow-up analyses included: (1) evaluation of genetic correlation with brain-related phenotypes; (2) estimation of predicted genetically driven differential expression in brain tissues; (3) colocalization of genetic association loci with cis-eQTLs; (4) evaluation of loci pleiotropy, and (5) druggability of nominated targets.

This study provides an unequivocal GWS association signal for the intron 1 locus in *OPRM1* and, through haplotype analysis, suggests that rs1799971 (A118G) may not be the driver of the locus’s association with OA. We also identified two novel GWS gene-based associations with OA: *PPP6C* and *FURIN*. Both genes have been previously associated with phenotypes correlated with OA (e.g. *PPP6C* with cigarette smoking^31,32^, alcohol consumption^31^, and depressive symptoms^33^; *FURIN* with schizophrenia^34,35^, risk tolerance^36^, and insomnia^36^). This study links these genes to predicted genetically driven differential expression in brain tissues by OA. Colocalization analysis supports a shared single variant between OA association and gene expression for *PPP6C* but the results for *OPRM1* and *FURIN* are not as well defined. Collectively, these results provide extended insight into the association of *OPRM1* with OA and implicate novel genes associated with this phenotype.

## Results

### Different approaches to defining OA are highly genetically correlated

Our gSEM for OA brings together novel GWAS data from GENOA and summary statistics from all prior GWAS of OA that included more than 1000 cases and 1000 controls of European ancestry (EA)^26–28^. GENOA is a new consortium comprised of investigators who attend the National Institute on Drug Abuse Genetics and Epigenetics Cross-cutting Research Team Meetings and who have GWAS data on OA (Supplementary Table 1). In this study, OA refers to a broad meaning of addiction to opioids defined by multiple approaches to measuring the phenotype. The success of both the GENOA and gSEM analyses to maximize sample size and discovery then depends on similar heritability and high genetic correlations across the different measures of OA.

We focused on EA cohorts for the genetic correlation and gSEM analyses because these approaches, which allow us to maximize sample size by bridging phenotypes and accounting for cohort overlap, require linkage disequilibrium score regression (LDSC) results to model the genetic variance–covariance matrix. LDSC, in turn, depends on an ancestry-specific reference panel, which isn’t currently available for African Americans (AAs).

Among the 10 independent EA cohorts contributing to GENOA, OA was defined by Diagnostic and Statistical Manual criteria for opioid abuse or dependence (DSM-based; N = 17,061) or by frequency of use (FOU) of illicit opioids (e.g., injecting heroin 10 or more times in the past 30 days; FOU-based; N = 11,976; Supplementary Table 1). SNP-based heritability for both phenotypes was strong (DSM-based: *h*^2^ = 0.11, *SE* = 0.03; FOU-based: *h*^2^ = 0.18, *SE* = 0.04) and their genetic correlation robust (*r*_*g*_ = 1.05, *SE* = 0.16; SNP-based genetic correlations are not bound by 1.0).

Across the full set of GWAS results contributing to the gSEM GWAS (i.e., GENOA, MVP-SAGE-YP, PGC-SUD, and PH) there are additional OA definitions (MVP and PH used Electronic Health Record ICD-9 or ICD-10 codes for opioid use disorder [OUD]) and variation in type of controls used. GENOA cohorts used a combination of controls (opioid exposed, unexposed, and unknown exposure public controls). MVP and PH used opioid exposed controls, and the PGC-SUD results used here were based on unexposed controls. Regardless of the approach to defining OA or the type of controls, the LDSC genetic correlations across cohorts were very high (all pairwise *r*_*g*_ > 0.9, Supplementary Table 2). These heritabilities and genetic correlations show that the genetics contributing to OA are highly shared regardless of OA case definition or opioid exposure status of controls to which the cases are compared.

### GENOA GWAS identifies one European Ancestry specific OA association

Conducting ancestry specific and cross-ancestry meta-analyses of the GENOA cohorts (Supplementary Figs. 1–6 and Supplementary Tables 3–5) yielded one GWS association locus on chromosome 4 among EAs (rs28386916-A, beta = 0.17, *p* = 9.04 × 10^–9^). The rs28386916 variant was not associated with OA among AAs (beta = − 0.025, *p* = 0.51) and consequently was no longer significant in the EA + AA meta-analysis. Rs28386916-A is common (EUR MAF = 0.40; AFR MAF = 0.81) and was well imputed (imputation quality > 0.8 across cohorts). However, it is an intronic variant located between (T)_11 and (A)_11 simple repeats within the long noncoding RNA ENST00000659878, which may undermine confidence in this result (Supplementary Fig. 7).

### Genomic structural equation model GWAS of opioid addiction identifies two other GWS loci in European Ancestry

**Figure 1:**
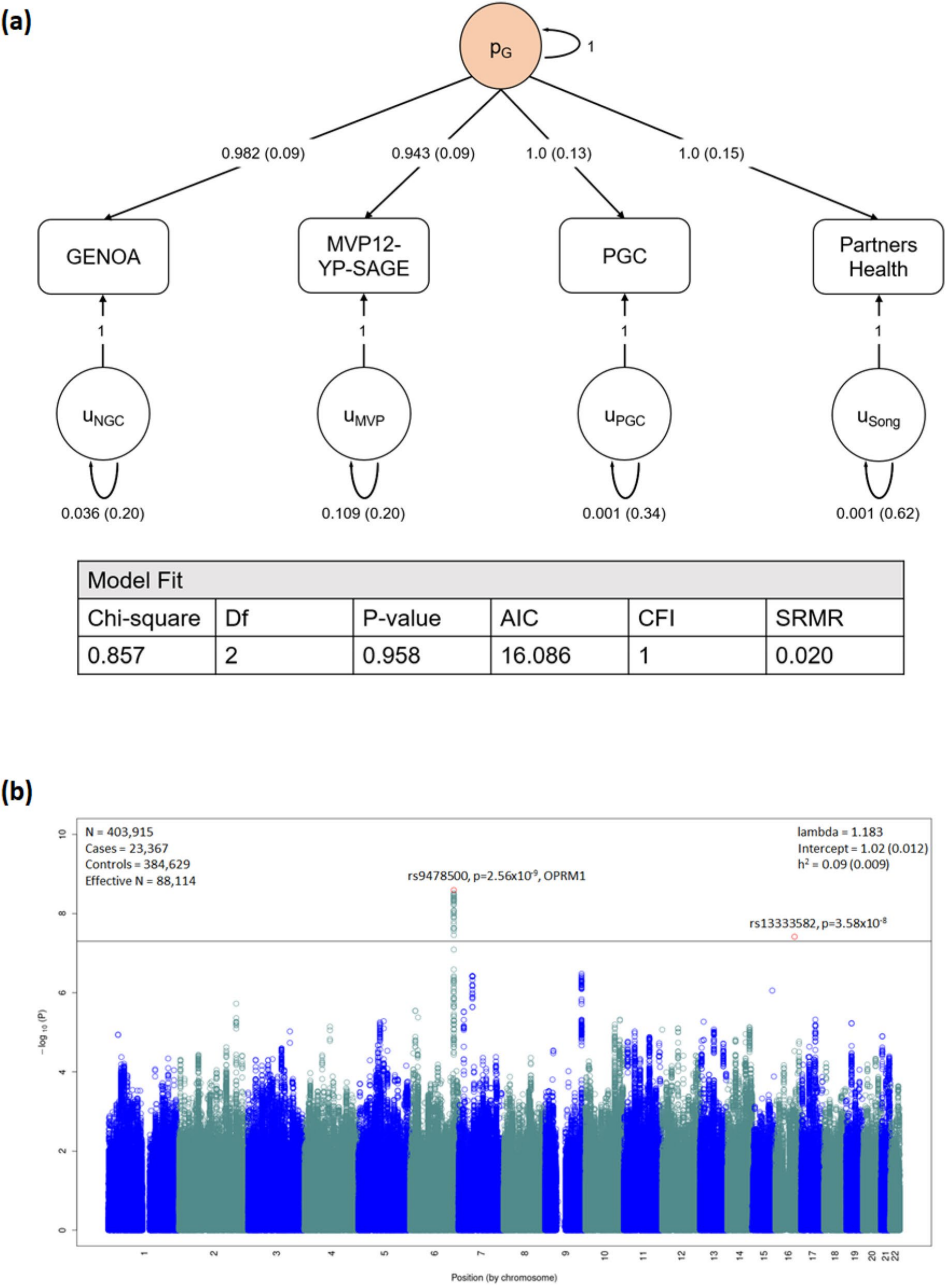
Genomic SEM model and Manhattan plot. (**a**) A common factor (p_g_) gSEM model (using GenomicSEM) is fit with summary statistics from GENOA, MVP12-YP-SAGE, PGC, and Partners Health cohorts. Standardized estimates and standard errors are shown for each free parameter. Model fit is shown by a non-significant chi-square test, high Akaike information criterion (AIC, higher is better) and comparative fit index (CFI) equal to 1.0, and low standardized root mean squared root (SRMR) values (ideally < 0.05). (**b**) Manhattan plot for gSEM results with summary statistics from GWAS from each cohort. Bonferroni correction was used to correct for multiple comparisons; associations with P < 2 × 10^–8^ (indicated by horizontal black bar) were genome-wide significant (top SNP highlighted in red).

A single common factor gSEM (Fig. 1a) fit the GENOA, MVP-SAGE-YP, PGC-SUD, and PH summary statistics for OA well, with high Akaike information criterion and comparative fit index, and low standardized root mean squared root (SRMR) values (Fig. 1a). Testing the association of 2.4 million variants available across all cohorts (Supplementary Tables 6 and 7 for LDSC and gSEM SNP QC and derivation of available variants, respectively) with the latent genetic factor (effective sample size N = 88,114) identified two GWS loci (Fig. 1b; Q–Q plot Supplementary Fig. 8): one on chromosome 6 with 32 GWS variants (top variant rs9478500-C, beta = 0.136, *p* = 2.56 × 10^–9^; Supplementary Table 8; forest plot Supplementary Fig. 9a) and the other represented by a single variant on chromosome 16 (rs13333582-C, beta = − 0.219, *p* = 3.58 × 10^–8^; forest plot Supplementary Fig. 10, LocusZoom plot Supplementary Fig. 12). The LDSC intercept of approximately 1 for this model (Fig. 1b), indicates that these results are not due to uncontrolled inflation, as would be expected if the overlap in the cohorts contributing to some of the summary statistics used here was not adequately accounted for^30^.

The associated locus on chromosome 6 was centered in intron 1 of the mu-opioid receptor gene *OPRM1* (Supplementary Fig. 11). The minor allele of the lead variant, rs9478500-C, was associated with increased risk of OA (beta = 0.136). All of the GWS variants were in high linkage disequilibrium (LD) with each other (r^2^ > 0.88 and D’ > 0.93; Supplementary Table 9). The previously reported missense variant rs1799971 (*OPRM1*-A118G), which was GWS for OUD in MVP-SAGE-YP^27^, was less statistically significant in our gSEM analysis (rs1799971-G, beta = − 0.115, *p* = 1.94 × 10^–6^; forest plot Supplementary Fig. 9b). In the MVP GWAS rs9478500-C was associated with OA, but with less statistical significance (MVP rs9478500-C, beta = 0.09, *p* = 4.31 × 10^–5^).

Analyses of rs9478500 conditioning on rs1799971 and vice versa using GCTA-COJO^37,38^ showed only modest decrements in statistical significance for each variant compared to their unconditional models: rs9478500 (p = 2.56 × 10^–9^ for the gSEM analysis vs. p = 2.04 × 10^–7^ for the conditional analysis); rs1799971 (p = 1.94 × 10^–6^ for the gSEM analysis vs. p = 1.41 × 10^–4^ for the conditional analysis). These results suggest that these variants have largely independent associations. However, GCTA-COJO only accounts for LD in terms of r^2^, which is low between these variants among those of European ancestry (r^2^ = 0.035). In contrast, perfect D’ (1.0) between these variants indicates a strong dependency in the haplotype structure and a need to examine haplotypes.

**Figure 2:**
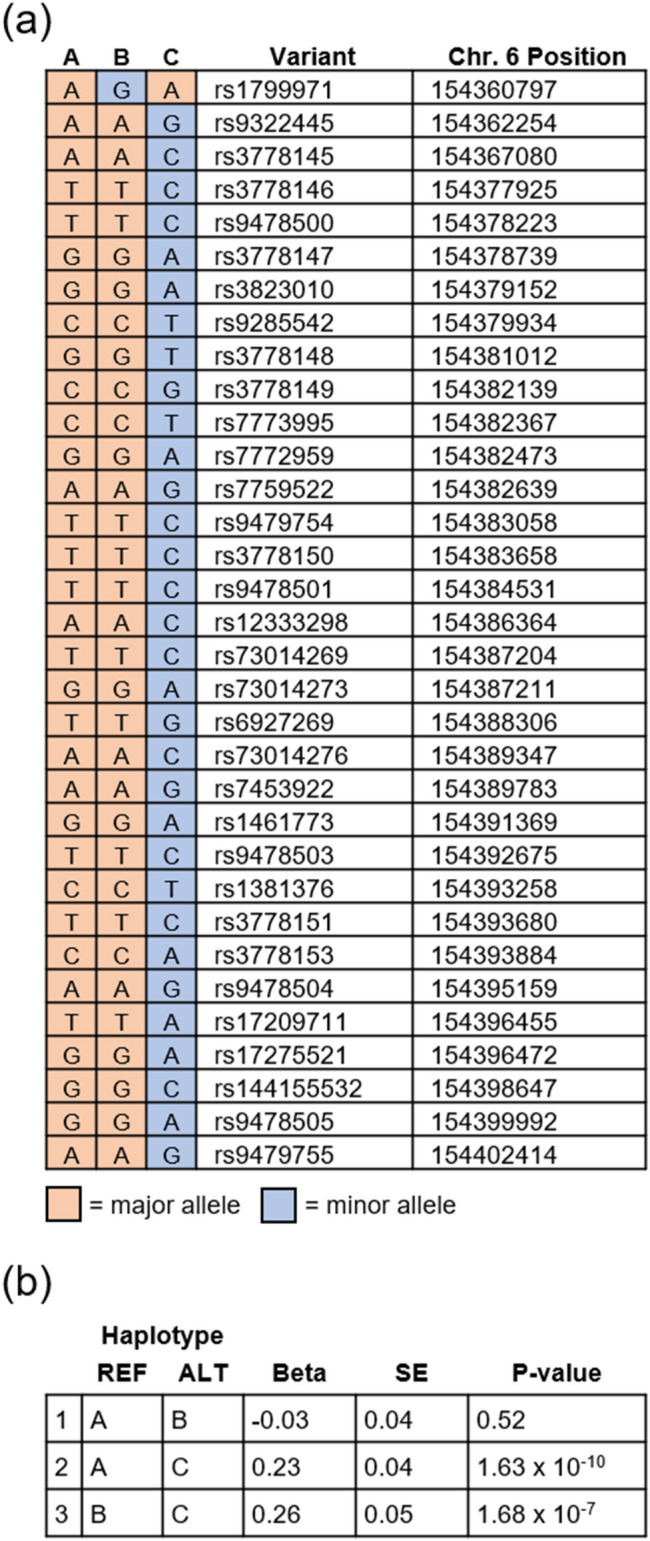
Association of major haplotypes for genome-wide significant *OPRM1* variants with OA. (**a**) The 3 major haplotypes for genome-wide significant *OPRM1* variants. Haplotype A is the predominant haplotype (frequency ~ 0.69 among contributing cohorts) and consists of major alleles for all variants. Haplotype B (frequency ~ 0.13 among contributing cohorts) consists of the minor allele for rs1799971 and the major allele for all other variants. Haplotype C (frequency ~ 0.16 among contributing cohorts) consists of the major allele for rs1799971 and minor allele for all other variants. The cohorts for whom we had the raw data to conduct the haplotype analyses were: UHS, VIDUS, ODB, Yale-Penn, CATS and Kreek (Supplementary Table 1). (**b**) Association of *OPRM1* haplotypes with OA. Haplotype C is associated with increased risk of OA when compared to Haplotype A or Haplotype B, whereas Haplotype B does not have a significant impact on OA relative to Haplotype A. The single variant results using the cohorts contributing to the haplotype analyses were: rs1799971 beta = -– 0.058, *p* = 0.135; rs9478500 beta = 0.205, *p* = 2.43 × 10^–9^.

Prior candidate gene studies that examined *OPRM1* haplotypes with rs1799971 suggested that other variants may explain its equivocal association with OA^39,40^. Subject level genotypes for haplotype analysis were available from a subset of cohorts contributing to the gSEM analysis (Fig. 2). In this subset of cohorts, the single variant results for rs9478500 (beta = 0.205, *p* = 2.43 × 10^–9^) were strong, but those for rs1799971 were weak (beta = − 0.058, *p* = 0.135). Comparison of the three haplotypes formed by rs1799971, rs9478500, and the other GWS *OPRM1* variants (Fig. 2a) further weakened evidence for an association with OA being driven by rs1799971 (Fig. 2b comparison 1: *p* = 0.52, beta = − 0.026, SE = 0.0397) and strengthened evidence for an association with OA being driven by the effect of the non-rs1799971 variants (comparison 2: *p* = 1.63 × 10^–10^, beta = 0.2303, SE = 0.036, Fig. 2b).

Intergenic variant rs13333582 on chromosome 16 was also GWS (Supplementary Fig. 12), with the minor C allele decreasing risk of OA (beta = − 0.22). While rs13333582 passed QC, (MAF = 0.04, INFO > 0.8, for all cohorts), variants in strong LD showed weaker evidence for association (e.g., rs921982 r^2^ = 0.87, p = 2.25 × 10^–3^). In addition, rs13333582 was not significant in the GENOA AA analysis (p = 0.28), and the direction of effect was opposite of that observed in the gSEM EA analysis.

The GWS association locus on chromosome 4 from the GENOA EA analysis (rs28386916-A, beta = 0.17, *p* = 9.04 × 10^–9^) was not included in the gSEM analysis as it was absent from the MVP and PH GWAS. However, several variants in high LD (D’ > 0.6) with it were not significant in the gSEM analysis (minimum p-value for rs11943738—p = 0.001, beta = 0.057, and SE = 0.017, Supplementary Table 10).

### OA is genetically correlated with 21 other brain-related traits

**Figure 3:**
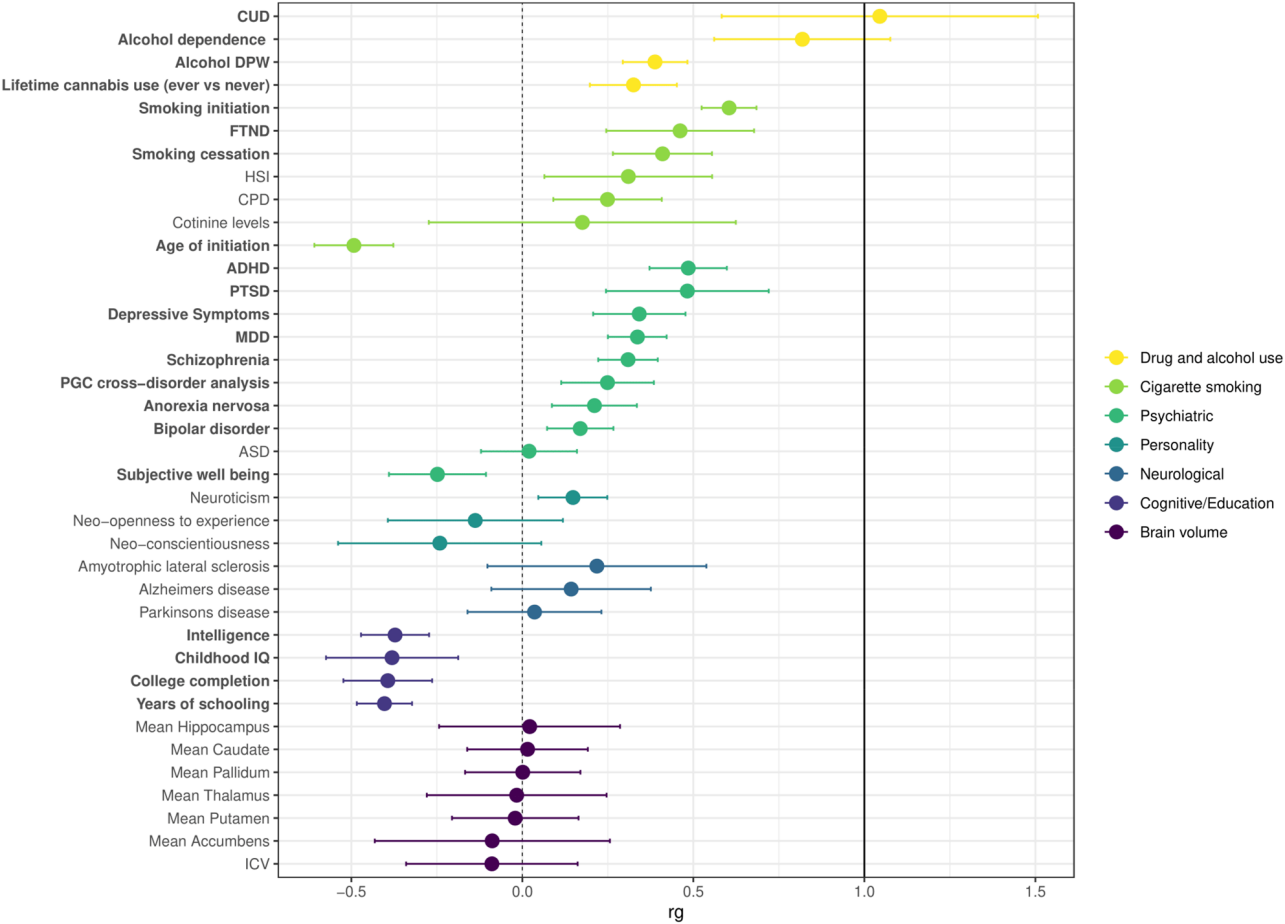
Genetic correlations of opioid addiction (OA) with 38 other brain-related phenotypes. Correlations were calculated using linkage disequilibrium (LD) score regression with the gSEM OA GWAS meta-analysis results, compared with results made available via LD Hub or study investigators (see Supplementary Table 24 for original references). Phenotypes were grouped by disease/trait or measurement category, as indicated by different colorings. Dots indicate the mean values for genetic correlation (*r*_g_); error bars show the 95% confidence intervals; the dashed vertical black line corresponds to *r*_g_ = 0 (no correlation with OA), and the solid vertical black line corresponds to *r*_g_ = 1.0 (complete correlation with OA). Phenotypes with significant correlation with OA are bolded (1 degree of freedom Chi-square test; Bonferroni adjusted *p*-value < 0.05 after accounting for 38 independent tests). Exact *p*-values are provided in Supplementary Table 10). CUD,  Cannabis use disorder; DPW,  drinks per week; FTND, Fagerström test for nicotine dependence; HSI, heaviness of smoking index; CPD, cigarettes per day; ADHD, attention deficit hyperactivity disorder; PTSD, post-traumatic stress disorder; MDD, major depressive disorder; ASD,  autism spectrum disorder; ICV,  intracranial volume.

LDSC-based genetic correlation (r_G_) analyses between OA (gSEM results) and 37 brain related traits (Fig. 3, Supplementary Table 11) yielded 21 significant results (Bonferroni threshold of *p* < 1.35 × 10^–3^). The highest r_G_s were with cannabis use disorder (r_G_ = 1.0452, p = 9.40 × 10^–6^) and alcohol dependence (r_G_ = 0.8183, p = 4.82 × 10^–10^); smoking traits (r_G_ = − 0.4928 to 0.6043, p = 1.82 × 10^–49^ to 0.44) and other psychiatric disorders (r_G_ = − 0.2482 to 0.4849, p = 0.79 to 1.82 × 10^–49^) were more modest. Expected inverse genetic correlations were also evident for age of initiation of cigarette smoking (r_G_ = − 0.4928, p = 5.97 × 10^–17^) and cognitive/educational traits (r_G_ = − 0.3722 to − 0.4033, p = 1.00 × 10^–4^ to 1.40 × 10^–22^). There were no genetic correlations between OA and brain volume traits.

### Gene-based MAGMA GWAS of gSEM summary statistics for OA corroborates *OPRM1* and identifies novel genes

**Figure 4:**
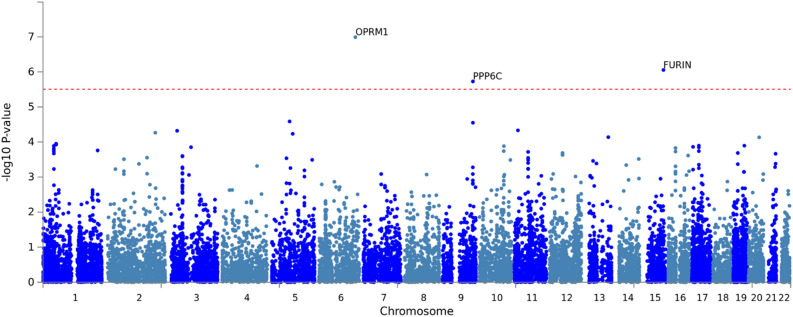
Gene-level Manhattan Plot. GWAS results were summarized at the gene-level using MAGMA. Bonferroni correction was used to correct for multiple comparisons; associations with P < 3 × 10^–6^ (indicated by horizontal red dotted line) were genome-wide significant.

Gene level analyses of gSEM GWAS results using MAGMA^41^ showed significant associations with *OPRM1* and two novel genes out of 15,977 genes tested (*p* < 3.13 × 10^–6^; Fig. 4; Q-Q plot Supplementary Fig. 13; full results in Supplementary Table 12). These novel gene-based associations include Protein Phosphatase 6 Catalytic subunit gene (*PPP6C*) and Furin Paired Basic Amino Acid Cleaving Enzyme gene (*FURIN*). The association between *PPP6C* and OA utilized 57 variants including several in LD that approached variant-level genome-wide significance (*p* = 3.37 × 10^–7^ to 2.14 × 10^–5^, Fig. 1b). This peak extended across three genes: *PPP6C*, *SCAI*, and *RABEPK* (Supplementary Fig. 14) but only PPP6C reached gene-based genome-wide significance (*SCAI p* = 0.0016; *RABEPK p* = 2.82 × 10^–5^).

The gene-level association between *FURIN* and OA was based on a single variant (rs17514846-A, beta = − 0.08, *p* = 8.82 × 10^–7^). While other *FURIN* variants were available in individual cohorts, the gSEM GWAS, and thereby the MAGMA analysis, included only one due to the gSEM method’s requirement that variants be present in every contributing cohort. A standard logistic regression meta-analysis of *FURIN* variants across the subset of GWAS cohorts without overlapping participants (GENOA, MVP, and PH) retained additional variants excluded from the gSEM analysis and identified 3 additional variants in strong LD with rs17514846 (r^2^ > 0.64, D’ = 1.0); all four variants were associated with OA (Supplementary Fig. 15; Supplementary Table 13), the weakest association being for rs17514846 (*p* = 1.67 × 10^–6^) and the strongest being for rs11372849, which was GWS (rs11372849-TC, beta = -0.074, *p* = 4.11 × 10^–8^; forest plot Supplementary Fig. 16).

### Predicted genetically driven gene expression in brain tissue expands neurobiologically relevant evidence for OA-associated genes

We applied S-PrediXcan^42^ using GTEx version 8 eQTL gene models (http://predictdb.org/) with the gSEM GWAS summary statistics as input to estimate genetically driven differential gene expression in human brain tissues associated with OA. Fourteen gene-tissue combinations surpassed correction for the total number of gene models and brain tissues (156,215 tests) with a false discovery rate (FDR) less than 0.05 (Table 1; all results presented in Supplementary Table 14). Predicted genetically driven *OPRM1* expression was significantly associated with OA in cerebellum. Only four brain tissues had gene models for *OPRM1* (cerebellum, cerebellar hemisphere, hypothalamus, and nucleus accumbens; Supplementary Table 14). In contrast, *PPP6C* was predicted to be differentially expressed in nine of 12 available brain tissue models. Nearby *SCAI* was the only other gene to show statistically significant genetically driven expression associated with OA, doing so across four brain tissues. *FURIN* was nominally associated (*p* = 9.67 × 10^–5^) in hippocampus but did not surpass the FDR < 0.05 threshold. *RABEPK* was not predicted to be differentially expressed by OA (best p = 0.055 in caudate).

**Table 1:** Fourteen gene-brain region combinations exhibiting predicted genetically driven differential gene expression in human brain regions associated with OA (across tissue FDR < 0.05) in analysis of gSEM GWAS summary statistics with S-PrediXcan analysis using GTEx version 8 eQTL gene models.

Gene	Tissue	Across tissue FDR
*OPRM1*	Cerebellum	0.009
*SCAI*	Cerebellum	0.009
*SCAI*	Frontal cortex	0.009
*SCAI*	Hippocampus	0.009
*PPP6C*	Hippocampus	0.009
*PPP6C*	Anterior cingulate cortex	0.01
*PPP6C*	Cerebellar hemisphere	0.01
*PPP6C*	Putamen basal ganglia	0.01
*PPP6C*	Caudate basal ganglia	0.01
*SCAI*	Cortex	0.01
*PPP6C*	Cortex	0.01
*PPP6C*	Frontal cortex	0.01
*PPP6C*	Hypothalamus	0.01
*PPP6C*	Nucleus accumbens basal ganglia	0.01

### Some OA associations colocalize with genetically driven gene expression

**Figure 5:**
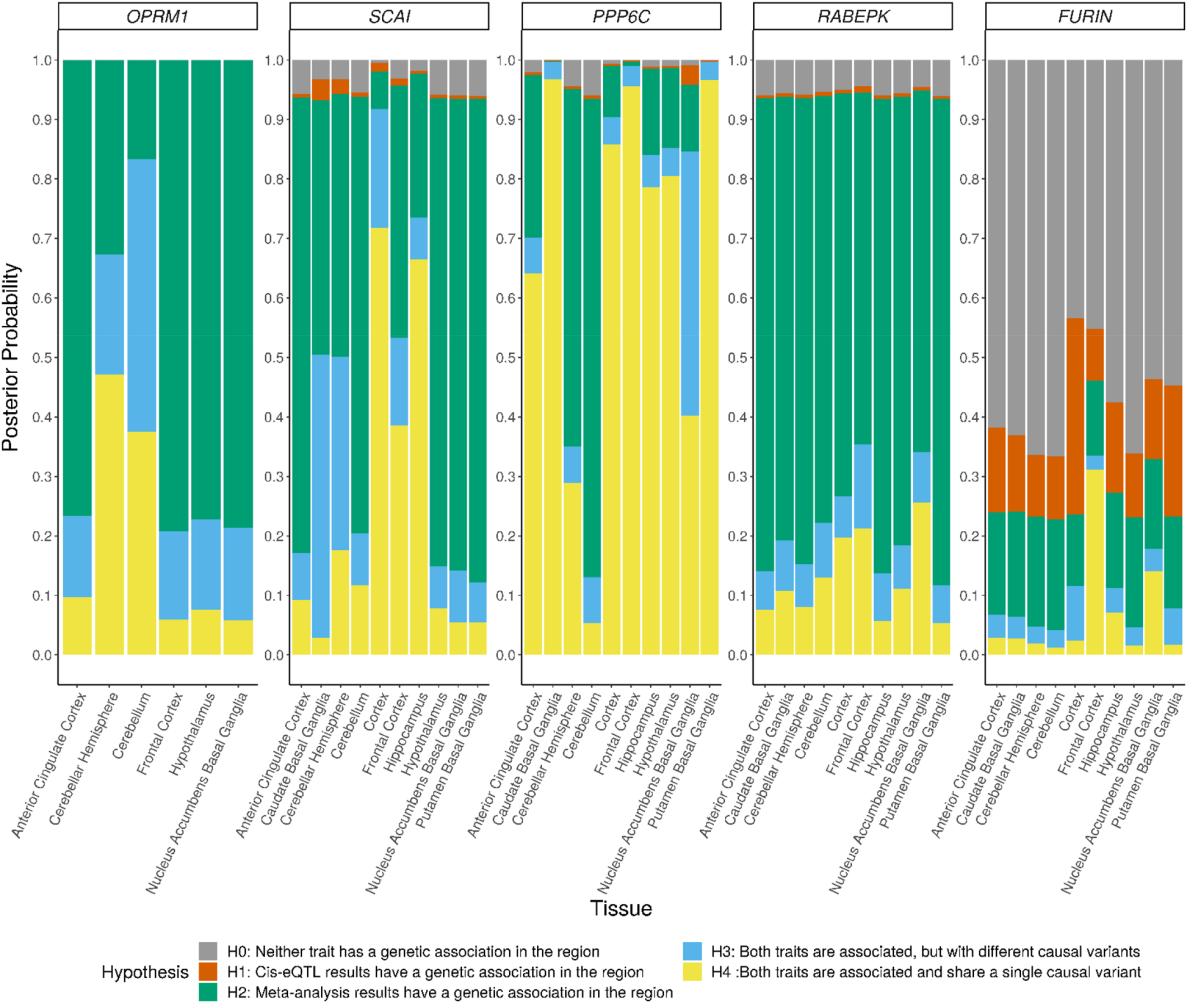
Colocalization of GWAS loci and QTLs for selected genes across 10 brain tissues. Posterior probabilities of supporting hypotheses regarding the association of each trait with SNPs in a region were calculated using coloc. For *OPRM1*, SNP-gene cis-eQTL associations were reported in GTEx Analysis v8 for only 6 of the 10 tissues.

To estimate the likelihood that the genetic loci associated with OA share a causal variant with the expression quantitative trait loci (eQTLs) for our nominated genes (*OPRM1, PPP6C,* and *FURIN*), we applied coloc^43^ to our gSEM GWAS results and the GTEx eQTL results for these genes. Because the variants underlying the GWS association for *PPP6C* physically extend into *SCAI* and *RABEPK* (Supplementary Fig. 14), we included these genes in the analysis. We evaluated colocalization across the superset of 10 brain tissues which showed genetically driving differential expression for at least one gene in the S-PrediXcan analysis (Supplementary Table 14). *OPRM1* is expressed at relatively low levels in the GTEx brain tissues (Supplementary Fig. 17a). Only six of 10 brain tissues showed variant associations with *OPRM1* expression in GTEx and could be included in the coloc analysis. The posterior probabilities for four tissues of the six tissues tested for *OPRM1* favored the hypothesis that only a genetic association with OA at this locus is present (Fig. 5H2, Supplementary Table 15). However, in the cerebellum, where *OPRM1* is most highly expressed and for which S-PrediXcan predicted differential expression by OA, the greatest posterior probabilities favored hypotheses for both the OA-associated locus and *cis*-eQTL traits being associated, but with different causal variants (H3) or a shared single causal variant (H4). Among the three genes at the *PPP6C*-centered locus, *PPP6C* shows the highest levels of gene expression in brain tissues (Supplementary Fig. 17b–d) and the greatest support for colocalization of OA-associated variants with *cis*-eQTLs for *PPP6C* (Fig. 5, Supplementary Table 15). In contrast, the analysis for *RABEPK* uniformly indicated that the OA-associated variants do not colocalize with the *RABEPK cis*-eQTLs. The analyses for *SCAI* showed mixed results, indicating the OA-associated variants have a moderate probability of colocalization with *SCAI* cis-eQTLs for some tissues (cortex, frontal cortex, and hippocampus) but not for most, and not as high a probability as for *PPP6C*. For *FURIN*, the null hypothesis of neither trait being associated in this region has the highest posterior probability across all tested brain tissues (Fig. 5, Supplementary Table 15), which is consistent with (1) a single variant (rs17514846) driving the GWS gene-based association with OA, (2) no significant evidence for differential gene expression in the S-PrediXcan analyses, (3) limited evidence for this variant as an eQTL in brain tissues (Supplementary Table 16).

### Drug repurposing analyses suggest druggability for *OPRM1*, *PPP6C*, and *FURIN*

To identify potential for new pharmacological treatments of OA through drug repurposing or compound development, we examined *OPRM1*, *PPP6C*, and *FURIN* across multiple drug repurposing databases (the Drug Gene Interaction Database v.3.0 [DGIdb]^44^, Connectivity Map [CMap]^45^, PHAROS [https://pharos.nih.gov/]^46^). *OPRM1* is a known target of more than one-hundred drugs and compounds, including illicit drugs, abused therapeutics (e.g., oxycodone), and OA treatments (e.g., methadone and buprenorphine) (Supplementary Table 17a–c). In contrast, *PPP6C* is not a target of any known drug or compound but has a 94% likelihood that its protein has ligand properties based on its chemistry^47^. *FURIN* is the target of one approved drug, pirfenidone, which is indicated for treatment of idiopathic pulmonary fibrosis. There are more than 80 compounds identified targeting *FURIN*, most developed as inhibitors targeting *FURIN* function in infectious diseases (Supplementary Table 18a–c).

### Testing previously reported GWAS associations supports three variants & two genes: rs1799971, rs62103177, rs640561, *BEND4, and PTPRF*

Among the previously reported associations, the strongest association in the current results was observed for rs1799971 (*p* = 1.94 × 10^–6^, see Supplementary Table 20 for previously GWAS associated variants and genes)^27^. The only other previously reported variant tested in our gSEM was rs62103177 in the *KCNG2* gene^23^, which was nominally associated with OA in our EA cohort (*p* = 0.0024). To capture variants reported to be association with OA that were not included in the gSEM results, we extended our lookup to standard logistic regression meta-analysis of the subsets of EA GWAS cohorts that were not included in the originally reported findings. The *CNIH3* variant, rs10799590, reported by Nelson et al.^24^ and the PGC-SUD reported^48^ variant, rs201123820, were not statistically significant in our standard meta-analysis (p = 0.49 and p = 0.63, respectively). Of the two PH reported variants^28^, only rs10014685 was present in independent cohorts, but it was not significant in either (deCODE p = 0.89; UHS p = 0.36). Examining a recently reported GWAS of prescription opioid misuse (POU)^49^, we see a genetic correlation between OA and POU (r_g_ = 0.74, *p* = 2.24 × 10^–12^) and extend their association of rs640561 to OA (rs640561-T, beta = -0.061, *p* = 0.009). Finally, we examined our gene-based GWAS results for evidence supporting previously reported genes and found no support for *GRM8*^22^ (*p* = 0.655), *CNIH3*^24^ (*p* = 0.174), *CCDC42*^50^ (*p* = 0.307) or *SPDYE4*^50^ (p = 0.856). However, we found nominal support for *BEND4* (*p* = 0.0023) association with OA, which was reported in the PGC-SUD GWAS for the opioid use phenotype (exposed vs. unexposed controls)^26^ and *PTPRF* for POU (*p* = 0.026)^49^.

## Discussion

In this study, we demonstrated a high degree of genetic correlation between differing diagnostic and frequency-based case definitions of OA and across different types of controls (opioid exposed, unexposed, and public controls), which allowed us to conduct the GENOA meta-analysis and apply gSEM successfully to GENOA and existing summary statistics to conduct the largest GWAS among European ancestry cohort participants to date (23,367 cases and total effective sample size of 88,114 individuals) with the OA case definition. The GENOA GWAS of European ancestry identified a single GWS association (rs28386916), but the variant was not available in the MVP or PH cohorts and, consequently, was not tested for replication and was not present in the gSEM GWAS. Given the position of this variant between simple repeats and that variants in high LD with it were not significant in the gSEM GWAS, it seems likely that it represents a false positive. In the gSEM GWAS we found the strongest statistical evidence to date linking variants in intron 1 of the *OPRM1* gene to OA, extending previous candidate gene studies focused on this gene^39,51,52^. Gene-based analyses also identified two novel GWS genes for OA: *PPP6C* and *FURIN*. Examining the predicted differential expression of these genes in brain tissue and their colocalization with OA association signals suggest that the effect of the *PPP6C* locus on risk of OA is likely to be through effects on *PPP6C* expression, while the signal for *OPRM1* is more complex; there is limited evidence that expression differences explain the association of *FURIN* with OA.

The top finding of this gSEM GWAS for OA was centered in intron 1 of the *OPRM1* gene (lead SNP rs9478500-C). Prior candidate gene studies of this region have found nominal associations of variants in intron 1, including some of those that are GWS here (e.g., rs1381376, rs3778151, & rs3778150)^39,51,52^. The mu-opioid receptor gene, *OPRM1*, has long been a target of OA research and drug development. The functional coding variant rs1799971 (A118G; Asn40Asp) has been studied at length with equivocal results^39,53,54^. In the current GWAS era, only the MVP GWAS of OUD found rs1799971 to be GWS (*p* = 1.51 × 10^–8^)^27^. Adding cohorts to the MVP summary statistics in the current study reduced the variant’s association with OA to *p* = 1.94 × 10^–6^.

Conditional analyses using GCTA-COJO^37,38^ to evaluate the independence of rs9478500 and rs1799971 suggested that each association was independent of the other. However, GCTA-COJO only accounts for LD in terms of r^2^ which is low between these variants in EA (0.035), whereas D’ is 1.0. Thus, following prior candidate gene studies^39,40^, we examined the associations of specific *OPRM1* haplotypes with OA. The OA association was strongest with the haplotype consisting of the major rs1799971-A allele and minor alleles at all GWS *OPRM1* variants from our study, which was associated with increased risk compared to the other haplotypes. The association with the haplotype consisting of the minor rs1799971-A allele and major alleles at all other GWS *OPRM1* variants was not significant. In the subset of cohorts used in the haplotype analysis, the variant level association for rs1799971 was also not significant, which limits the strength of our haplotype-based conclusions. However, our earlier haplotype analyses of *OPRM1* intron 1 variants and rs1799971 came to similar conclusions, albeit in more limited datasets^39,40^. This relationship between the underlying EA haplotype structure and risk for OA may explain the equivocal findings at the individual rs1799971 variant level, but it may be that other, as-yet-unidentified variants could be the true causal variants driving these haplotype associations. Given the GCTA-COJO conditional analysis, the haplotype analysis, and the potential for as-yet-unidentified variant the exact nature of this locus’ association with OA remains to be determined.

The role of genetically driven *OPRM1* expression also appeared complex in this study. In our S-PrediXcan analysis, we observed statistically significant, predicted differential expression of *OPRM1* for OA in cerebellum, the brain tissue with the highest level of *OPRM1* expression in GTEx. Moreover, one of the two *cis*-eQTL variants in the version 8 GTEx model for *OPRM1* expression (rs478498) is in high LD with our top association variant (rs9478500; r^2^ = 0.56, D’ = 0.98), suggesting that the intron 1 locus may have its effect on OA through *OPRM1* expression. However, the colocalization analysis was more equivocal. The hypotheses with the greatest posterior probabilities were that both *OPRM1* expression and OA risk are associated with this locus, but with different causal variants (H3, *posterior probability* = 0.46) and with a single causal variant (H4, *posterior probability* = 0.38). Given the generally low level of *OPRM1* expression across bulk brain tissues, larger sample sizes and single nuclei experiments will be needed to further distinguish which of these hypotheses is most likely. Ultimately, model organism or organoid experiments are likely to be necessary to fully test gene expression as a potential mechanism for the association of this locus with OA.

Beyond *OPRM1*, we also observed a GWS association with OA for the intergenic variant rs13333582. The observations that variants in high LD with rs13333582 had much weaker associations and that rs13333582 is located in a repetitive sequence both suggest the association with OA was a false positive.

Increasing statistical power through a gene-based GWAS of the gSEM summary statistics identified two novel GWS genes for OA: *PPP6C* and *FURIN*. *PPP6C* (Protein Phosphatase 6 Catalytic subunit gene) is a component of a signaling pathway that regulates cell cycle progression known to be involved with the immune system and cancer (https://www.uniprot.org/uniprot/O00743#function). However, the gene is also strongly expressed in a variety of adult human brain tissues (Supplementary Fig. 17c) and is linked to abnormal locomotor behavior in mice (http://www.informatics.jax.org/diseasePortal/genoCluster/view/20628^56^). Predicted biological processes for *PPP6C* include G-protein coupled purinergic nucleotide receptor signaling pathway (GO:0035589) (https://maayanlab.cloud/archs4/gene/PPP6C), which affects regulation of neurons, microglia and astrocytes^57^. Predicted genetically driven differential expression of *PPP6C* by OA was significant across several brain regions, and colocalization analysis of *PPP6C cis*-eQTLs and the OA-variant association signal at this locus also showed a high probability of being driven by a shared single variant. Because the *PPP6C*-centered association locus extends into the nearby genes *SCAI* and *RABEPK*, and significant predicted genetically driven differential expression of *SCAI* was also observed, we cannot exclude the possibility that these other genes play a role in, or are responsible for, the *PPP6C*-OA association. However, the degree of gene expression/variant association colocalization for *PPP6C* across brain tissues suggest it as the leading candidate for follow-up studies.

The GWS gene-based association of OA with *FURIN* was driven by a single variant, rs17514846. However, this signal was supported by analysis of additional *FURIN* variants in a subset of cohorts where more *FURIN* variants were available, including a GWS association with OA at the variant level for rs11372849. *FURIN* is a member of the convertase family and encodes a type 1 membrane bound protease that is expressed in neuroendocrine and brain tissues, among others (https://www.ncbi.nlm.nih.gov/gene/5045). Although *FURIN* shows higher expression across brain tissues than *OPRM1*, the S-PrediXcan analysis did not show significant predicted genetically driven expression differences associated with OA for this gene, and the highest posterior probabilities from the colocalization analysis favored no association of either eQTLs or the OA-variant association signal at this locus. However, this may reflect the single variant association with OA in the gSEM GWAS and an effect on OA through mechanisms other than gene expression.

LDSC analyses demonstrated moderate to strong genetic correlations between OA and a variety of substance use, psychiatric, and cognitive phenotypes in expected directions (e.g., positive correlation with cannabis use disorder, inverse correlation with age of smoking initiation). Moving from the general genomic signal to the specific OA-associated genes, we observed important differences in *OPRM1*, *PPP6C*, and *FURIN* associations with brain- and SUD-related phenotypes. Although *OPRM1* has been broadly studied, only GWAS of OUD^58^ and methadone dose^59^ have identified GWS associations with *OPRM1* variants (Supplementary Fig. 18; Supplementary Table 19a)^60,61^. The variant associated with methadone dose, rs73568641, was not associated with OA in this gSEM GWAS (*p* = 0.328). In contrast with *OPRM1*, variants in *PPP6C* have been associated with numerous brain- and SUD-related phenotypes, notably opioid medication use, alcohol consumption, numerous smoking phenotypes, and depression among others (Supplementary Fig. 19; Supplementary Table 19b). The specific *PPP6C* variants nominally associated with OA in this gSEM GWAS have been associated with neuroticism, depressive symptoms and a number of smoking phenotypes at genome-wide significance (Supplementary Table 19b). Variants in *FURIN* have been associated with other brain- and SUD-related phenotypes, most predominantly schizophrenia, but also risk taking, number of sexual partners, and insomnia (Supplementary Fig. 20; Supplementary Table 19c). The variant driving the GWS gene-based result here (rs17514846) and the GWS variant in our subset meta-analysis for OA (rs11372849) are associated with each of these phenotypes at genome-wide significance (Supplementary Table 19c).

Similar variability was seen across *OPRM1*, *PPP6C*, and *FURIN* in drug repurposing analyses. While *OPRM1* is the known target of more than 100 drugs and compounds, *FURIN* is the target of one approved drug (pirfenidone), and *PPP6C* is not a target of any known drug or compound However, *FURIN* is the target of more than 80 compounds and the *PPP6C* protein has a 94% likelihood of being ligandable. Should these novel gene associations be validated in subsequent studies they appear to be potentially important drug development targets.

Among this study’s limitations, the most notable is the focus on cohorts of European ancestry. This focus was required to maximize sample size and statistical power by combining summary statistics across GENOA, PGC-SUD, MVP and PH GWAS through application of the gSEM GWAS method. This approach allowed us to more than double the number of cases used in the EA-focused MVP-SAGE-YP analyses that yielded genome-wide significance for rs1799971 (n = 23,367 vs n = 10,544) by leveraging gSEM’s ability to model multiple correlated phenotypes and account for sample overlap. These gSEM analyses will be extended to African Americans when the needed ancestry-specific LDSC reference panel becomes available.

An additional potential limitation is the variability in OA case definitions (e.g., diagnostic and frequency of use) and types of controls (e.g., exposed, unexposed, and population-based) used to define OA phenotypes across the cohorts within the GENOA and across the other contributing GWAS. However, the genetic correlations across phenotypes were uniformly high (*r*_*g*_ > 0.9) and resulted in a well-fitting single latent factor gSEM model. An important caveat to the high correlations observed across cohorts with exposed, unexposed, and public controls is that the exposure to opioids was often based on prescribed medication (MVP and PH), which differs in risk of OA from exposure to illicit heroin use. Fine-grained comparison of large samples with different types of exposure to opioids will be needed to resolve this question. Because we have incorporated GENOA and previously published GWAS of OA for our discovery analyses, we do not have independent replication cohorts available in which to test the identified associations. However, OA associations with the intron 1 locus have been previously reported^29,39,51,52^, the chromosome 16 (rs13333582) is an eQTL for a gene previously reported as associated with OA^55^, and variants in both *PPP6C* and *FURIN* have previously been associated with substance use and psychiatric disorder traits that are highly associated with OA (Supplementary Figs. 12 and 13). Thus, independent replication remains to be demonstrated, but the available evidence supports the identified variants and genes as associated with OA.

In this study, we leveraged gSEM across new and existing OA GWAS that employed various opioid-related phenotypes to conduct the largest EA-focused GWAS to date. Our results show the strongest statistical evidence to date for an association between variants in intron 1 of *OPRM1* with OA. Conflicting results from variant conditional analysis and haplotype analysis of the GWS variants and previously associated rs1799971 (A118G) indicate that further functional study of intron 1 variants will be needed to determine the causal variant(s) driving this association with OA. Gene-based analyses identified two GWS associations: *PPP6C* and *FURIN*. These genes are novel for OA, however, variants within them have been associated at genome-wide significance with related phenotypes, such as cigarette smoking, alcohol consumption, general risk taking, and schizophrenia. With strong SNP-based heritability for these OA phenotypes, but only these few GWS findings, it is clear that increased sample sizes for GWAS and complementary approaches (e.g., gene regulation in postmortem brain tissues) are needed to identify much of the genetics driving risk of OA as well as to extend these studies to non-European ancestries.

## Methods

### Ethics declarations

All contributing cohorts obtained necessary patient/participant consent and appropriate institutional IRB approval received. Meta-analyses of these genetic data is approved under RTI International IRB review (IRB ID: CR00000710 for 14107). All methods were performed in accordance with the relevant guidelines and regulations.

### Cohorts and opioid addiction phenotype

Descriptive statistics for the GENOA studies contributing previously unpublished GWAS of opioid addiction (OA) to this investigation are provided in Supplementary Table 1, and full descriptions of the studies are provided in the Supplementary Methods. In total, this analysis provides new OA GWAS results for 304,831 individuals, including 7281 cases and 297,550 controls, with an effective sample size of 28,428 (4/((1/# Cases) + (1/# Controls)))^62,63^.

In the GENOA studies, OA was defined based either on the frequency of opioid use (FOU) or Diagnostic and Statistical Manual (DSM) of Mental Disorders criteria. Some studies included only opioid-exposed individuals in their control groups, while others included both exposed and unexposed individuals.

### Genotype quality control and imputation

Sites in the GENOA consortium conducted standard genotype quality control using filters appropriate for their samples. SNPs were filtered based on call rate and deviation from Hardy–Weinberg equilibrium. Samples were filtered based on call rate, excessive homozygosity, relatedness, and sex discrepancies. Classification of European ancestry individuals was based on comparison to reference populations using STRUCTURE^64^. Specific filters used for each sample are provided in Supplementary Table 22.

For most samples, genotype imputation was performed with the Michigan Imputation Server^65^ using the 1000 Genomes Phase 3 v5 reference panel. For COGA, genotypes were phased with SHAPEIT2^66^ and imputed with Minimac3^65^ using the 1000 Genomes Phase 3 v5 reference panel. For deCODE, genotype imputation was conducted by long-range phasing and haplotype imputation of chip-genotyped individuals with methods described previously^67^.

### Association testing

Imputed genotypes for GENOA studies were tested for association with opioid addiction case–control status, adjusting for sex, age, genotype principal components and in some cases recruitment site and other study-specific covariates. For all cohorts except deCODE, association testing was performed using the score test meta-analysis model of rvtests^68^. For COGA, which is a family study, an empirically determined kinship matrix was used with rvtests to account for relatedness. For deCODE, data were analyzed using logistic regression treating disease status as the response and imputed genotype counts as covariates. Other available individual characteristics that correlate with disease status were also included in the model as nuisance variables (sex, age, county of origin), blood sample availability, and an indicator function for the overlap of the lifetime of the individual with the time span of phenotype collection) using previously described methods^69^. To account for inflation due to population stratification and relatedness, test statistics were divided by an inflation factor (1.10) estimated from linkage disequilibrium score regression (LDSR)^70^.

GENOA cohorts were combined via inverse variance-weighted meta-analysis of variants with MAF > 0.01 and Rsq > 0.8 using METAL^71^ with genomic control enabled. For characterization of FURIN, an additional inverse variance-weighted meta-analysis of chromosome 15 was conducted including all studies with no overlapping samples (GENOA + MVP without SAGE and Yale-Penn + Partners; Cases N = 16,849, Controls N = 379,493, Total N = 396,342, Effective Total N = 52,508).

### Cohort descriptions for GenomicSEM

The gSEM analysis includes results on EA from the GENOA meta-analysis, Million Veteran Program (MVP), Psychiatric Genetics Consortium Substance Use Disorders Group (PGC-SUD), and the Partners Health cohorts. The MVP results are based on a meta-analysis including MVP parts 1 and 2 (total of 8529 OUD cases and 71,200 opioid-exposed controls), along with Yale-Penn and Study of Addiction: Genetics and Environment (SAGE) cohorts (total of 10,544 OUD cases and 72,163 opioid-exposed controls)^27^. The PGC-SUD results include 4,503 opioid dependence cases and 4173 unexposed controls^26^. Unexposed controls are used for the PGC-SUD because the exposed controls results have negative heritability estimates. The partners health cohort includes 1,039 OUD cases and 10,743 exposed controls^28^. Note that the GENOA GWAS, Yale-Penn, and SAGE parts of the MVP, and the PGC-SUD results include overlapping samples. However, accounting for this sample overlap is a feature of the gSEM approach applied in this study. A total of 2,434,903 variants were present in all cohorts and tested for association with the OA latent variable in the gSEM analysis using a total sample size of 403,915 (23,367 cases and 384,619 controls; effective sample size of 88,114).

### Genomic structural equation modeling

The GenomicSEM package^30^ within R was used for genomic structural equation modeling (gSEM). The gSEM implemented multivariable LDSC function within this package was used to calculate single-nucleotide polymorphism heritability on the observed and liability scale (prevalence of 10%), genetic covariance matrices, and genetic correlation. The LD scores from 1000 Genomes Project phase 3 European^70^ were used as the reference population in this calculation. The sampling genetic covariance matrix is expanded to incorporate SNP effects by including the covariances between SNPs and each cohort. This expanded sampling genetic covariance includes the multivariate LDSC estimated genetic variances and covariances, along with the sampling covariance matrix of the SNP effects on the cohorts, which are estimated using cross-trait LDSC with the sampling correlation weighted by the sample overlap. With the gSEM implemented LDSC, the overlap of samples between GENOA, MVP meta-analysis, and PGC-SUD is not a concern.

A single latent factor gSEM was used with the residual variance of the latent factor set to 1 to normalize the loading estimates. The loadings were calculated using diagonally weighted least squares and residual variances were bound to above 0.01 to avoid negative residual variance estimates.

### Post-GWAS QC

Standard post-GWAS QC was performed on the association results for individual cohorts, the GENOA meta-analysis results, and the gSEM results. For the association results for individual cohorts, the following QC was performed on the summary statistics: (1) variants with MAF < 0.01, imputation quality < 0.8, missing values, or values outside expected ranges were removed; (2) variant IDs were standardized; (3) QQ plots were generated to confirm lack of excessive bias and genomic control was applied to remove residual bias (see Supplementary Table 23 for genomic control factors); and (4) LDSC was performed to confirm correlation among the different OA measures used by cohorts (Supplementary Table 2). For the meta-analysis and gSEM results, the following QC steps were performed: (1) QQ plots were generated to confirm lack of excessive bias and (2) GWS signals were examined for consistency of effect size and direction of effect across cohorts.

### Conditional analyses

To assess the interdependence of OPRM1 signals, GCTA-COJO^37,38^ was used to conduct conditional analyses (using the --cojo-cond option to specify the variant to condition on) based on the summary statistics from the gSEM analysis. Separate analyses conditioning on rs1799971 and rs9478500 were performed. UHS was used as the genotype reference for these analyses.

### Haplotype analyses

To conduct haplotype analyses raw genotype data is needed. Chromosome 6 genotypes were available from UHS, VIDUS, ODB, Yale-Penn, CATS and Kreek (Supplementary Table 1). These cohorts’ data were phased with Eagle v2.4 via the Michigan Imputation Server^65^. Haplotypes for samples from each study were constructed by extracting *OPRM1* variants that were GWS in the gSEM analysis and concatenating ordered by genomic position. Supplementary Table 24 provides counts for the various haplotypes observed across the studies. The 3 most common haplotypes, which accounted for 98% of observed haplotypes, were tested for association with OA in R adjusting for sex and genotype principal components. Only individuals carrying exclusively these haplotypes were included in the analysis. Two models were run, one in which the haplotype containing all major alleles served as the reference haplotype and one in which the haplotype containing the minor rs1799971-G allele served as the reference. This approach provided three effective comparisons: (a) rs1799971-G haplotype versus the major allele haplotype; (b) minor allele+rs1799971-A haplotype versus the major allele haplotype; (c) minor allele+rs1799971-A haplotype versus rs1799971-G haplotype. Individual cohort results were combined in an inverse variance-weighted meta-analysis using METAL (N = 21,037).

### Gene-based analyses

Gene-based associations with OA were calculated from the gSEM summary statistics with MAGMA v1.08^41^ with a 10 kb gene window via the Functional Mapping and Annotation (FUMA) of GWAS web tool v1.3.6a^72^. The gSEM summary statistics were mapped to 15,977 protein coding genes, resulting in a Bonferroni-corrected threshold of p = 3.129e-6 for declaring genome-wide significance.

### Cross-trait genetic correlations with OA

Summary statistics from the gSEM were used as input into LD score regression (LDSC) with reference to the 1000 Genomes EUR panel to estimate genetic correlations between OA and 38 other complex phenotypes. These phenotypes were categorized into the following groups: drug and alcohol use, cigarette smoking, psychiatric, personality, neurological, cognitive/education, and brain volume. The full list of phenotypes and GWAS datasets, as obtained from LD Hub or shared by the original investigators, are provided in the Supplementary Table 25.

### PrediXcan

To investigate the transcriptome-wide associations between predicted gene expression and OA, we employed the MetaXcan v0.6.6^42^ method. Briefly, MetaXcan uses association summary statistics to predict associations between gene expression and a phenotype of interest association. Gene expression models were predicted from tissue-specific eQTL datasets. To increase the performance of our prediction models, we used the MASHR-M^73^ models built on fine-mapped variables from DAP-G^74^. The specific models we used were pre-computed MetaXcan models available through PredictDB (http://predictdb.org/) for 12 brain regions (Supplementary Table 14) that were generated using the GTEx^75^ version 8 datasets.

Summary level statistics from the Genomic SEM analysis were used as input to MetaXcan. Prior to input, summary statistics were harmonized according to the best practices guide outlined on the MetaXcan wiki. As part of this process, the gwas_parsing.py utility (https://github.com/hakyimlab/summary-gwas-imputation) was used to lift summary statistics over to the human genome build version 38 and provide harmonized variant identifiers compatible with those used by GTEx v8. To increase the number of overlapping markers between our summary statistics and the fine-mapped pre-built MASHR models, we imputed missing summary associations as suggested by the best practices workflow. Imputation was performed separately for each chromosome using the gwas_summary_imputation.py utility (https://github.com/hakyimlab/summary-gwas-imputation) and the pre-computed parquet genotype, genotype metadata files, and European LD block files available through the MetaXcan zenodo repository^76^. Imputed summary statistics were finally re-combined using the gwas_summary_imputation_postprocess.py utility.

The resulting imputed, harmonized association summary statistics were then used as input to MetaXcan. The number of genes tested for each tissue is found in Supplementary Table 14. FDR correction was applied to account for the number of genes tested across all tissues (156,215 total tests). A gene’s predicted expression was considered significantly associated with OA if its FDR-adjusted p-value fell below a threshold of 0.05.

### Colocalization

Co-localization analysis was performed using the coloc package in R^77^. Cis-eQTL data for individuals of European ancestry from the GTEx v8 eQTL Tissue-Specific All SNP Gene Associations dataset (dbGaP Accession phs000424.v8.p2) were input as a quantitative trait into coloc (sample sizes for each tissue type indicated in Supplementary Table 15). Summary statistics from the gSEM analysis were input as a quantitative trait, with a sample size of 403,915. Summary statistics from the standard meta-analysis of OA were input as a case–control trait with 16,849 cases and 379,493 controls. All SNP positions were lifted over to build 38. The cis-eQTL data was partitioned into blocks based on the gene in the SNP-gene pair. For each gene block, only SNPs in the gSEM or meta-analysis summary statistics overlapping with the cis-eQTL data were input into the coloc function for (approximate) Bayes Factor colocalization analysis.

## Supplementary Information

Supplementary Information 1.

Supplementary Tables.

## Data Availability

The GWAS summary statistics generated and/or analyzed during the current study will be made available via dbGAP; the dbGaP accession assigned to the UHS is phs000454.v1.p1. The website is https://www.ncbi.nlm.nih.gov/projects/gap/cgi-bin/study.cgi?study_id=phs000454.v1.p1.

## Code availability

Docker images and WDL workflows for analyses of primary GENOA data and gSEM meta-analysis are available at the following Github repositories: https://github.com/RTIInternational/biocloud_docker_tools; https://github.com/RTIInternational/biocloud_gwas_workflows; https://github.com/RTIInternational/biocloud_wdl_tools.

## References

[1] Ahmad, F.B., Rossen, L.M. & Sutton, P. Provisional drug overdose death counts. (National Center for Health Statistics, 2021).

[2] Rudd RA, Aleshire N, Zibbell JE, Gladden RM (2016). Increases in drug and opioid overdose deaths - United States, 2000–2014. MMWR Morb. Mortal Wkly. Rep..

[3] National Safety Council. In *Motor vehicle deaths estimated to have dropped 2% in 2019* (2020).

[4] Substance Abuse and Mental Health Services Administration. In *Key substance use and mental health indicators in the United States: Results from the 2019 National Survey on Drug Use and Health (HHS Publication No. PEP20-07-01-001, NSDUH Series H-55)* (Center for Behavioral Health Statistics and Quality, Substance Abuse and Mental Health Services Administration., Rockville, MD, 2020).

[5] Florence CS, Zhou C, Luo F, Xu L (2016). The economic burden of prescription opioid overdose, abuse, and dependence in the United States, 2013. Med. Care.

[6] Leslie DL, Ba DM, Agbese E, Xing X, Liu G (2019). The economic burden of the opioid epidemic on states: The case of Medicaid. Am. J. Manag. Care.

[7] National Academies of Sciences, Engineering, and Medicine. In *Medications for opioid use disorder save lives* (eds Leshner, A. & Mancher, M.) (Washington D.C., 2019). 30896911

[8] Koob GF, Volkow ND (2010). Neurocircuitry of addiction. Neuropsychopharmacology.

[9] Kreek MJ (2012). Opiate addiction and cocaine addiction: Underlying molecular neurobiology and genetics. J. Clin. Invest..

[10] Santiago-Rivera OJ, Havens JR, Parker MA, Anthony JC (2018). Risk of heroin dependence in newly incident heroin users. JAMA Psychiat..

[11] Vowles KE (2015). Rates of opioid misuse, abuse, and addiction in chronic pain: A systematic review and data synthesis. Pain.

[12] Kendler KS, Jacobson KC, Prescott CA, Neale MC (2003). Specificity of genetic and environmental risk factors for use and abuse/dependence of cannabis, cocaine, hallucinogens, sedatives, stimulants, and opiates in male twins. Am. J. Psychiatry.

[13] Goldman D, Oroszi G, Ducci F (2005). The genetics of addictions: Uncovering the genes. Nat. Rev. Genet..

[14] Gatz M (2006). Role of genes and environments for explaining Alzheimer disease. Arch. Gen. Psychiatry.

[15] Klaver CC (1998). Genetic risk of age-related maculopathy. Population-based familial aggregation study. Arch. Ophthalmol..

[16] Zaitlen N (2014). Leveraging population admixture to characterize the heritability of complex traits. Nat. Genet..

[17] Levran O, Yuferov V, Kreek MJ (2012). The genetics of the opioid system and specific drug addictions. Hum. Genet..

[18] Hancock DB, Markunas CA, Bierut LJ, Johnson EO (2018). Human genetics of addiction: New insights and future directions. Curr. Psychiatry Rep..

[19] Crist RC, Reiner BC, Berrettini WH (2019). A review of opioid addiction genetics. Curr. Opin. Psychol..

[20] Gelernter J, Polimanti R (2021). Genetics of substance use disorders in the era of big data. Nat. Rev. Genet..

[21] Nielsen DA (2008). Genotype patterns that contribute to increased risk for or protection from developing heroin addiction. Mol. Psychiatry.

[22] Nielsen DA (2010). Genome-wide association study identifies genes that may contribute to risk for developing heroin addiction. Psychiatr. Genet..

[23] Gelernter J (2014). Genome-wide association study of opioid dependence: Multiple associations mapped to calcium and potassium pathways. Biol. Psychiatry.

[24] Nelson EC (2016). Evidence of CNIH3 involvement in opioid dependence. Mol. Psychiatry.

[25] Cheng Z (2018). Genome-wide association study identifies a regulatory variant of RGMA associated with opioid dependence in European Americans. Biol. Psychiatry.

[26] Polimanti R (2020). Leveraging genome-wide data to investigate differences between opioid use vs. opioid dependence in 41,176 individuals from the Psychiatric Genomics Consortium. Mol. Psychiatry.

[27] Zhou H (2020). Association of OPRM1 functional coding variant with opioid use disorder: A genome-wide association study. JAMA Psychiat..

[28] Song W (2020). Genome-wide association analysis of opioid use disorder: A novel approach using clinical data. Drug Alcohol Depend..

[29] Nelson EC (2014). Association of OPRD1 polymorphisms with heroin dependence in a large case-control series. Addict Biol..

[30] Grotzinger AD (2019). Genomic structural equation modelling provides insights into the multivariate genetic architecture of complex traits. Nat. Hum. Behav..

[31] Liu M (2019). Association studies of up to 1.2 million individuals yield new insights into the genetic etiology of tobacco and alcohol use. Nat. Genet..

[32] Wootton RE (2020). Evidence for causal effects of lifetime smoking on risk for depression and schizophrenia: A Mendelian randomisation study. Psychol. Med..

[33] Baselmans BML (2019). Multivariate genome-wide analyses of the well-being spectrum. Nat. Genet..

[34] Schizophrenia Working Group of the Psychiatric Genomics, C. Biological insights from 108 schizophrenia-associated genetic loci. *Nature* **511**, 421–7 (2014). 10.1038/nature13595 PMC4112379 25056061

[35] Periyasamy S (2019). Association of schizophrenia risk with disordered niacin metabolism in an Indian genome-wide association study. JAMA Psychiat..

[36] Karlsson-Linner R (2019). Genome-wide association analyses of risk tolerance and risky behaviors in over 1 million individuals identify hundreds of loci and shared genetic influences. Nat. Genet..

[37] Yang J, Lee SH, Goddard ME, Visscher PM (2011). GCTA: A tool for genome-wide complex trait analysis. Am. J. Hum. Genet..

[38] Yang J (2012). Conditional and joint multiple-SNP analysis of GWAS summary statistics identifies additional variants influencing complex traits. Nat. Genet..

[39] Hancock DB (2015). Cis-expression quantitative trait loci mapping reveals replicable associations with heroin addiction in OPRM1. Biol. Psychiatry.

[40] Levran O, Awolesi O, Linzy S, Adelson M, Kreek MJ (2011). Haplotype block structure of the genomic region of the mu opioid receptor gene. J. Hum. Genet..

[41] de Leeuw CA, Mooij JM, Heskes T, Posthuma D (2015). MAGMA: Generalized gene-set analysis of GWAS data. PLoS Comput. Biol..

[42] Barbeira AN (2018). Exploring the phenotypic consequences of tissue specific gene expression variation inferred from GWAS summary statistics. Nat. Commun..

[43] Giambartolomei C (2018). A Bayesian framework for multiple trait colocalization from summary association statistics. Bioinformatics.

[44] Cotto KC (2018). DGIdb 3.0: A redesign and expansion of the drug-gene interaction database. Nucleic Acids Res..

[45] Subramanian A (2017). A next generation connectivity map: L1000 platform and the first 1,000,000 profiles. Cell.

[46] Sheils TK (2021). TCRD and Pharos 2021: Mining the human proteome for disease biology. Nucleic Acids Res..

[47] Mitsopoulos C (2021). canSAR: Update to the cancer translational research and drug discovery knowledgebase. Nucleic Acids Res..

[48] Polimanti R (2020). Leveraging genome-wide data to investigate differences between opioid use vs opioid dependence in 41,176 individuals from the Psychiatric Genomics Consortium. Mol. Psychiatry.

[49] Sanchez-Roige S (2021). Genome-wide association study of problematic opioid prescription use in 132,113 23 and Me research participants of European ancestry. Mol. Psychiatry.

[50] Kalsi G (2016). Genome-wide association of heroin dependence in Han Chinese. PLoS ONE.

[51] Zhang H (2006). Association between two mu-opioid receptor gene (OPRM1) haplotype blocks and drug or alcohol dependence. Hum. Mol. Genet..

[52] Levran O (2008). Genetic susceptibility to heroin addiction: A candidate gene association study. Genes Brain Behav..

[53] Schwantes-An TH (2016). Association of the OPRM1 variant rs1799971 (A118G) with non-specific liability to substance dependence in a collaborative de novo meta-analysis of european-ancestry cohorts. Behav. Genet..

[54] Levran O, Kreek MJ (2020). Population-specific genetic background for the OPRM1 variant rs1799971 (118A>G): implications for genomic medicine and functional analysis. Mol. Psychiatry.

[55] Polimanti R (2021). Multi-environment gene interactions linked to the interplay between polysubstance dependence and suicidality. Transl. Psychiatry.

[56] Bult CJ (2019). Mouse genome database (MGD) 2019. Nucleic Acids Res..

[57] Zarrinmayeh HA-O, Territo PR (2020). Purinergic receptors of the central nervous system: Biology, PET ligands, and their applications. Mol. Imaging.

[58] Zhou H (2019). GWAS including 82,707 subjects identifies functional coding variant in OPRM1 gene associated with opioid use disorder. medRxiv.

[59] Smith AH (2017). Genome-wide association study of therapeutic opioid dosing identifies a novel locus upstream of OPRM1. Mol. Psychiatry.

[60] Ghoussaini M (2021). Open targets genetics: Systematic identification of trait-associated genes using large-scale genetics and functional genomics. Nucleic Acids Res..

[61] Ochoa D (2021). Open targets platform: Supporting systematic drug-target identification and prioritisation. Nucleic Acids Res..

[62] Marquez-Luna, C., Loh, P.R., South Asian Type 2 Diabetes, C., Consortium, S.T.D. & Price, A.L. Multiethnic polygenic risk scores improve risk prediction in diverse populations. *Genet. Epidemiol.* **41**, 811–823 (2017). 10.1002/gepi.22083 PMC5726434 29110330

[63] Holland D (2020). Beyond SNP heritability: Polygenicity and discoverability of phenotypes estimated with a univariate Gaussian mixture model. PLoS Genet..

[64] Pritchard JK, Stephens M, Donnelly P (2000). Inference of population structure using multilocus genotype data. Genetics.

[65] Das S (2016). Next-generation genotype imputation service and methods. Nat. Genet..

[66] Delaneau O, Howie B, Cox AJ, Zagury JF, Marchini J (2013). Haplotype estimation using sequencing reads. Am. J. Hum. Genet..

[67] Kong A (2008). Detection of sharing by descent, long-range phasing and haplotype imputation. Nat. Genet..

[68] Zhan X, Hu Y, Li B, Abecasis GR, Liu DJ (2016). RVTESTS: An efficient and comprehensive tool for rare variant association analysis using sequence data. Bioinformatics.

[69] Price AL (2009). The impact of divergence time on the nature of population structure: An example from Iceland. PLoS Genet..

[70] Bulik-Sullivan BK (2015). LD Score regression distinguishes confounding from polygenicity in genome-wide association studies. Nat. Genet..

[71] Willer CJ, Li Y, Abecasis GR (2010). METAL: Fast and efficient meta-analysis of genomewide association scans. Bioinformatics.

[72] Watanabe K, Taskesen E, van Bochoven A, Posthuma D (2017). Functional mapping and annotation of genetic associations with FUMA. Nat. Commun..

[73] Urbut SM, Wang G, Carbonetto P, Stephens M (2019). Flexible statistical methods for estimating and testing effects in genomic studies with multiple conditions. Nat. Genet..

[74] Wen X, Lee Y, Luca F, Pique-Regi R (2016). Efficient integrative Multi-SNP association analysis via deterministic approximation of posteriors. Am. J. Hum. Genet..

[75] Consortium TG (2015). The genotype-tissue expression (GTEx) pilot analysis: Multitissue gene regulation in humans. Science.

[76] Barbeira, A.N. & Im, H.K. GWAS summary statistics imputation support data and integration with PrediXcan MASHR. (ed. Zenodo) (2019).

[77] Giambartolomei C (2014). Bayesian test for colocalisation between pairs of genetic association studies using summary statistics. PLoS Genet..

